# The influencing factors of cancer-related fatigue in Chinese patients with myelodysplastic syndrome: A cross-sectional study

**DOI:** 10.1016/j.apjon.2025.100712

**Published:** 2025-05-02

**Authors:** Libing Liang, Jian Gao, Caiqin Wu, Yan Qian

**Affiliations:** a School of Nursing, Shanghai University of Traditional Chinese Medicine, Shanghai, China; b Nursing Department, Shanghai Sixth People's Hospital Affiliated to Shanghai Jiao Tong University School of Medicine, Shanghai, China; c Shanghai Jiao Tong University School of Nursing, Shanghai, China

**Keywords:** Myelodysplastic syndrome, Cancer-related fatigue, Influencing factor

## Abstract

**Objective:**

This study aimed to evaluate Cancer-related fatigue (CRF) prevalence in Myelodysplastic syndrome (MDS) and identify contributing factors. The goal was to aid in developing interventions to alleviate CRF, and refine clinical nursing practices.

**Methods:**

A cross-section study was conducted from June 2023 to April 2024, recruiting 144 MDS patients from a Shanghai hospital. Surveys and scales were used to assess fatigue, insomnia, caregiving burden, and nutritional status. Statistical analyses, including t-tests, ANOVA, correlation, and regression, were performed to identify CRF influencers.

**Results:**

The study revealed that the mean CRF score among patients with MDS was 28.63 ​± ​9.73, with a median score of 30 (IQR 22–35), indicating moderate CRF levels. CRF showed positive correlations with anxiety/depression, insomnia, nutritional status, and number of chemotherapies, while demonstrating a negative correlation with family support. Multivariate analysis identified age, chemotherapy regimen, Revised International Prognostic Scoring System (IPSS-R) risk category, anxiety/depression, and nutritional status as significant predictors of CRF.

**Conclusions:**

The study identified moderate CRF levels in patients with MDS, with severity influenced by age, IPSS-R risk category, chemotherapy regimen, anxiety/depression, and nutritional status. Multivariate analysis identified anxiety/depression and nutritional status as key contributing factors. These findings highlight the need for further research into the pathogenesis of MDS-associated CRF, particularly focusing on comprehensive assessment and targeted management of anxiety/depression and nutritional status in this patient population.

## Introduction

Myelodysplastic Syndromes (MDS), clonal myeloid malignancies originating from hematopoietic stem cell dysfunction, exhibit marked interpatient heterogeneity in clinical outcomes. Survival ranges from months to years, with approximately 30% progressing to Acute Myeloid Leukemia (AML).^1^ Age significantly influences incidence, escalating from 0.1/100,000 in patients under 40 years to 26.9/100,000 at 70–79 years, and peaking at 55.4/100,000 in octogenarians.^2^ Current risk-stratified therapies prioritize hematopoiesis restoration and quality-of-life (QoL) improvement in low-risk patients through erythropoiesis-stimulating agents, immunomodulators, transfusions, and iron chelation.^3^ High-risk patients receive hypomethylating agents (azacitidine/decitabine) and intensive chemotherapy, yet median survival remains poor (11–16 months), with allogeneic hematopoietic stem cell transplantation as the sole curative option.^4^ This highlights the urgent need to improve management methods.

Cancer-related fatigue (CRF), a multidimensional syndrome encompassing physical, emotional, cognitive exhaustion, represents a prevalent yet underaddressed complication affecting over 50% of MDS patients.^5^ This distressing symptom manifests as persistent subjective tiredness stemming from both disease processes and therapeutic interventions.^6^ Clinical urgency is compounded by treatment-exacerbated severity, particularly in elderly patients (> 70 years) who demonstrate increased susceptibility due to multimorbidity and treatment intolerance.^7^ The substantial socioeconomic impact is evident through treatment discontinuation rates (30% to 60%), employment limitations (50%), and care dependency (20%).^8^

The Theory of Unpleasant Symptoms (TOUS) provides a powerful framework for understanding the multifactorial nature of CRF. It posits that the influencing factors of symptoms consist of physiological, psychological, and environmental elements, emphasizing the dynamic interaction among these factors.^9^ Current mechanistic understanding reveals intricate pathways involving cytokine dysregulation, hypothalamic-pituitary-adrenal (HPA) axis dysfunction, circadian disruption, and mitochondrial abnormalities.^5^ Notably, 38% of low-risk MDS patients experience anxiety/depression—exceeding age-matched controls—which exacerbates neurotransmitter dysregulation and HPA dysfunction, fueling CRF progression.^10^ Social determinants, particularly family support, significantly influence CRF management. Robust family support mitigates perceived stress and enhances coping capacity, thereby alleviating CRF-associated psychological distress.^11^ A recent study examined the lagged relationships between sleep, fatigue, and depression during chemotherapy, revealing that objective insomnia, characterized by prolonged nighttime awakenings, predicted subsequent increases in fatigue, which in turn forecasted elevated depressive symptoms.^12^ Insomnia can influence fatigue through several pathways, including its effects on inflammatory activity^13^ or the disruption of circadian rhythms, immune dysfunction, and excessive cytokine production, all of which are independently related to fatigue.^5^ Furthermore, peripheral mechanisms such as mitochondrial dysfunction and ATP synthesis deficits may drive metabolic abnormalities in cancer patients.^5^ These factors, combined with the risks of malnutrition due to appetite loss and anemia/infection, significantly exacerbate fatigue symptoms.^14^ Evidence indicates that the incidence of CRF can be as high as 38% among patients experiencing malnutrition or weight loss, underscoring the critical role of nutritional status as a potential contributor to the onset or worsening of CRF.^15^

Despite global advancements in CRF characterization, critical knowledge gaps persist in three domains: 1) incomplete understanding of MDS-specific pathogenic mechanisms, 2) absence of population-specific evidence from mainland China, and 3) limited intervention studies hindered by disease heterogeneity and multifactorial fatigue etiology.^16^ This study employs the TOUS framework to systematically investigate CRF determinants in MDS patients, specifically examining physiological (insomnia, nutritional status), psychological (anxiety/depression), and environmental (family support) precursors. The findings aim to inform targeted intervention strategies addressing this debilitating symptom complex while advancing symptom management theory in hematological malignancies.

## Methods

### Design

This study employed a cross-sectional, observational, and correlational design, conducted at a single center. Adherence to the STROBE (Strengthening the Reporting of Observational Studies in Epidemiology) guidelines is ensured, with a detailed checklist provided in Supplementary materials.

### Participants

Between June 2023 and April 2024, participants were recruited via convenience sampling from the Hematology Department of Shanghai Sixth People's Hospital. Inclusion criteria comprised: (a) diagnosis of MDS per the Chinese Guidelines for Diagnosis and Treatment of Myelodysplastic Syndromes (2023 edition);^17^ (b) age ≥ 18 years; (c) absence of communication barriers between patients/caregivers and researchers; and (d) voluntary participation with written informed consent. Exclusion criteria included: (a) cognitive impairments or conditions precluding questionnaire completion; (b) comorbid systemic malignancies; (c) concurrent enrollment in interventional trials; or (d) Questionnaires with > 20% incomplete data were excluded from analysis.

Sample size estimation was based on a pre-survey indicating a 60% event probability (*P* ​= ​0.6). Using an allowable error margin (δ) of 0.15*P* and standard statistical methods for proportion estimation, the minimum required sample size was calculated as 114 participants. The study included 144 participants, providing adequate statistical power to investigate factors influencing CRF. A comprehensive participant enrollment flowchart is provided in Supplementary Fig. S1 to enhance methodological transparency and mitigate selection bias concerns.

### Measure

#### General information questionnaire (Appendix 1)

Data were collected using a researcher-designed structured questionnaire encompassing three domains: demographic characteristics (age, gender, marital status, education level, and monthly income), clinical characteristics, and laboratory parameters. Clinical characteristics included MDS subtype, IPSS-R risk category, disease duration, iron chelation therapy implementation, chemotherapy regimen, number of chemotherapies, and Eastern Cooperative Oncology Group (ECOG) performance status. Laboratory parameters comprised first-day hospitalization values of peripheral white blood cell count, hemoglobin concentration, platelet count, along with ferritin, albumin, and prealbumin levels, all retrieved from the hospital's electronic medical record system. Patients were categorized into low-risk (very low, low, and intermediate risk) and high-risk (high and very high risk) groups according to IPSS-R criteria.^18^ Study limitations include the relatively small sample size and predominant use of 5-Azacytidine and the CHG regimen (recombinant human granulocyte colony-stimulating factor ​+ ​homoharringtonine ​+ ​cytarabine), necessitating future studies with expanded cohorts and inclusion of diverse treatment regimens for comparative analysis.

Low-risk group patients hospitalized due to disease progression requiring transfusion-dependent status received supportive therapy, including granulocyte colony-stimulating factor (G-CSF) with prophylactic antibiotics to control neutropenia-related infections, platelet or packed red blood cell transfusions combined with tranexamic acid antifibrinolytic therapy to manage bleeding risks, and erythropoietin (EPO) with thalidomide to improve hematopoietic function.^17^ High-risk group patients were hospitalized for systemic chemotherapy (5-Azacytidine or CHG regimen [recombinant human granulocyte colony-stimulating factor ​+ ​homoharringtonine ​+ ​cytarabine]), hypomethylating agent therapy (HAM), or hematopoietic stem cell transplantation evaluation.^19^ This study established hemoglobin levels < 70 g/L or platelet counts < 50 ​× ​10^9^/L as the transfusion intervention threshold.^20^

Laboratory parameters were determined according to the Clinical and Laboratory Standards Institute (CLSI) C28-A3 guidelines^26^: the normal reference range for platelet count in adults is 150–450 ​× ​10^9^/L, with <100 ​× ​10^9^/L defined as significant reduction; white blood cell count normal reference range is 4.0–10.0 ​× ​10^9^/L, with < 4.0 ​× ​10^9^/L considered reduced; albumin normal range is typically 35–50 g/L, with < 30 g/L diagnostic of hypoalbuminemia; prealbumin normal reference range is 150–350 mg/L, with <150 mg/L indicating malnutrition. Iron metabolism indicators followed World Health Organization (WHO) criteria^21^: serum ferritin < 15 ng/mL was defined as iron deficiency, while > 1000 ng/mL required iron chelation therapy. Anemia classification adhered to WHO standards:^22^ hemoglobin > 130 g/L (female >120 g/L) was considered normal, 110–129 g/L (female 110–119 g/L) as mild anemia, 80–109 g/L as moderate anemia, and < 80 g/L as severe anemia.

#### Cancer fatigue scale (CFS) (Appendix 2)

The CFS was designed by Okuyama et al.^23^ and assess physical, emotional, and cognitive dimensions of CRF. It is composed of a total of 15 items rated from 0 to 4, where 0 points indicates “none at all”, 1 point indicates “very little”, 2 points indicates “a little”, 3 points denoted “quite a lot”, and 4 points signify “extremely much”. In this study, the Chinese - version of the CFS by Zhang et al., with good reliability and validity, was used.^24^ The CFS score ranges from 0 to 60, with higher scores meaning severer fatigue. In this study, the *Cronbach's α* coefficient of the scale was 0.906, and the *Cronbach's α* coefficients of each dimension were 0.726–0.893 respectively, indicating good reliability and validity. In the pre-experiment on MDS patients, patients with 14 or more points showed more prominent manifestations in multiple symptom dimension (physical, mental, and social aspects of fatigue), more limitations in daily activities, more severe sleep disorders, and lower quality of life overall.

#### Hospital anxiety and Depression Scale (HADS) (Appendix 3)

This scale is commonly used to screen the anxiety and depression levels of hospitalized patients. The Hospital Anxiety and Depression Scale (HADS) was developed by Zigmond AS and Snaith RP in 1983.^25^ This scale screened for anxiety (HADS-A: items 1, 3, 5, 7, 9, 10, 13) and depression (HADS-D: items 2, 4, 6, 8, 11, 12, 14) using 14 items scored 0–3. Subscale scores were interpreted as: 0–7 (normal), 8–10 (mild), 11–14 (moderate), and 15–21 (severe). In this study, the Cronbach's α coefficient of the scale was 0.901, and the *Cronbach's α* coefficients of each dimension were 0.820 and 0.834 respectively, showing good reliability and validity.

#### Insomnia severity index (ISI) (Appendix 4)

The ISI was developed by Morin et al.^26^ to screen insomnia and assess the nature and symptoms of sleep disorders of research subjects. This scale assessed sleep initiation, maintenance, quality, and related distress via seven items (0–4 scale). Total scores categorized insomnia severity as: 0–7 (none), 8–14 (mild), 15–21 (moderate), or 22–28 (severe). In this study, the *Cronbach's α* coefficient of the scale was 0.912, indicating good reliability and validity.

#### Family activity, pulse, grimace, appearance, respiration (APGAR) index (APGAR) (Appendix 5)

This questionnaire is a tool for assessing an individual's satisfaction with their family function. Zhang's Chinese adaptation^27^ measured family functional satisfaction using five Likert-scaled items (0–10 total). Scores were stratified as: ≥ 7 (good function), 4–6 (moderate impairment), and ≤ 3 (severe impairment). In this study, the *Cronbach's α* coefficient of this scale was 0.832, showing good reliability and validity.

#### Patient-generated subjective global assessment (PG-SGA) (Appendix 6)

It is the preferred nutritional assessment tool for cancer patients and is used to assess the nutritional status of MDS patients. This scale was modified and developed by Fauth.^28^ Its content includes body weight change, discomfort symptoms, appetite, physical condition, and physical examination. The first 4 parts are completed by patients, and the last 3 parts are evaluated by doctors. According to the patient's nutritional status, 0–3 points indicate normal nutrition; 4–8 points indicate mild malnutrition; and ≥ 9 points indicate severe malnutrition. In this study, the *Cronbach's α* coefficient of this scale was 0.856, demonstrating good reliability and validity.

### Data collection

Systematically trained researchers visited the hospital's hematology department to recruit eligible MDS patients. Potential participants received comprehensive details about the study's purpose and voluntary nature. Consenting individuals signed an informed consent form (Appendix 7) and were provided with a questionnaire requiring approximately 20–30 minutes to complete. Clinical data in the general information questionnaire were extracted from participants' electronic medical records, while physician evaluations were obtained for specific PG-SGA components. All other data collection occurred face-to-face, with participants granted sufficient time and privacy to complete the forms independently. Researchers addressed questions and provided assistance as needed. Upon completion, researchers verified all questionnaires to ensure data completeness.

### Data analysis

Statistical analyses were performed using IBM SPSS Statistics version 26.0. Normality of continuous variables was initially assessed through the Kolmogorov–Smirnov test, confirming normal distribution for key study variables. Descriptive statistics presented continuous variables as mean ​± ​standard deviation (SD) and categorical variables as frequencies (percentages). Independent *t*-tests and one-way ANOVA identified potential CRF influencing factors. Chi-square tests analyzed chemotherapy regimen distributions across IPSS-R risk stratifications to assess treatment rationality's impact on CRF. Subsequent one-way ANOVA with Tukey's HSD post-hoc testing compared CRF scores among different chemotherapy regimens. Pearson correlation analyses evaluated associations between CRF and anxiety/depression, insomnia, family support, subjective nutritional status, and number of chemotherapies. Variables demonstrating statistical significance (*P* ​< ​0.05) in univariate analyses were entered into a stepwise multiple linear regression model to quantify CRF risk factor contributions. All tests employed two-tailed analyses with α ​= ​0.05 significance threshold.

## Results

In this study, a total of 149 questionnaires were distributed and subsequently returned. Among them, 5 questionnaires were excluded due to more than 20% missing content, resulting in 144 valid questionnaires returned, with a valid return rate of 96.64%.

### Samples characteristics

A total of 144 MDS patients were included in this study. The average age was 61.33 ​± ​13.15 years old, and the average disease duration was 42.26 ​± ​53.86 months. The survey results indicated that among MDS patients, 54.9% were male and 45.1% were female; 41.7% were in the 60 to 69 age group; patients with RCMD accounted for 41.0%; and 75.7% had a normal body mass index (BMI). Further general data can be found in Table 1.

**Table 1:** Demographic characteristics and univariate analysis of latent profiles (*N* ​= ​144).

Characteristics	Number of cases	Cancer-related fatigue score	*t/F*	*P*-value
	*n* (%)	Mean ​± ​SD		
Sex			1.664^b^	0.098
Male	79 (54.9)	29.85 ​**±** ​9.33		
Female	65 (45.1)	27.15 ​**±** ​10.07		
Age (years)			38.979^c^	< 0.001∗∗∗
18-59	48 (33.3)	23.33 ​**±** ​1.49		
60-69	60 (41.7)	28.12 ​**±** ​0.97		
70-79	32 (22.2)	36.25 ​**±** ​1.14		
> ​80	4 (2.8)	39.00 ​**±** ​4.92		
Disease classification			5.674^c^	< 0.001∗∗∗
RA	11 (7.6)	19.73 ​**±** ​7.81		
RAS	17 (11.8)	25.59 ​**±** ​7.87		
RCMD	59 (41.0)	26.93 ​**±** ​10.19		
RAEB-I	30 (20.8)	32.73 ​**±** ​8.34		
RAEB-II	26 (18.1)	33.23 ​**±** ​7.99		
MDS-U	1 (0.7)	36.0 ​**±** ​0.00		
IPSS-R			5.639^a^	< 0.001∗∗∗
Low-risk group	83 (57.6)	25.17 ​**±** ​9.61		
High-risk group	61 (42.4)	33.34 ​**±** ​7.77		
Iron chelation therapy			−0.334^b^	0.739
No	115 (79.9)	28.50 ​**±** ​9.58		
Yes	29 (20.1)	29.17 ​**±** ​10.46		
Chemotherapy regimen				
5-Azacytidine			−4.453^b^	< 0.001∗∗∗
No	76 (52.8)	25.42 ​**±** ​10.04		
Yes	68 (47.2)	32.22 ​**±** ​8.04		
CHG regimen			−2.908^b^	0.004∗∗
No	86 (59.7)	26.74 ​**±** ​10.43		
Yes	58 (40.3)	31.43 ​**±** ​7.88		
5-Azacytidine ​+ ​CHG regimen			−3.311^b^	0.001∗∗
No	93 (64.6)	26.71 ​**±** ​10.12		
Yes	51 (35.4)	32.14 ​**±** ​7.94		
Whether to treat			4.215^a^	< 0.001∗∗∗
No	71 (49.3)	31.90 ​**±** ​8.02		
Yes	73 (50.7)	25.45 ​**±** ​10.24		
The number of chemotherapies (times)			17.617^c^	< 0.001∗∗∗
0	71 (49.3)	24.45 ​**±** ​9.47		
1-6	44 (30.6)	34.22 ​**±** ​8.29		
> 6	29 (20.1)	30.37 ​**±** ​7.55		
Serum ferritin level (ng/mL)			1.706^b^	0.090
Iron deficiency (< 15)	0 (0.0)	0.00 ​**±** ​0.00		
Normal (15–1000)	63 (43.8)	30.19 ​**±** ​9.24		
Iron overload (> 1000)	81 (56.3)	27.42 ​**±** ​9.98		
Peripheral WBC count level (× ​10^9^/L)			1.494^c^	0.228
Decrease (< 4.0)	95 (66.0)	29.82 ​**±** ​10.00		
Normal (4.0–10.0)	40 (27.8)	0.00 ​**±** ​0.00		
Increase (> 10.0)	9 (6.3)	27.06 ​**±** ​9.20		
Hemoglobin level (Male/Female, g/L)			0.573^c^	0.634
Normal (> 130/> 120)	21 (14.6)	28.52 ​**±** ​8.63		
Mild anemia (110–129/110-119)	26 (18.1)	27.23 ​**±** ​9.95		
Moderate anemia (80–109/80-109)	51 (35.4)	28.08 ​**±** ​10.13		
Severe anemia (< 80/< 80)	46 (31.9)	30.09 ​**±** ​9.75		
Platelet count level (× ​10^9^/L)			1.021^c^	0.363
Decrease (< 150)	117 (81.3)	29.15 ​**±** ​0.91		
Normal (150–450)	24 (16.7)	26.04 ​**±** ​1.92		
Increase (> 450)	3 (2.1)	29.00 ​**±** ​3.00		
Albumin level (g/L)			10.709^c^	< 0.001∗∗∗
Decrease (< 35)	36 (25.0)	34.17 ​**±** ​1.26		
Normal (35–50)	104 (72.2)	26.45 (0.95)		
Increase (> 50)	4 (2.8)	35.50 ​**±** ​1.19		
Prealbumin level (mg/L)			3.167^b^	0.002∗∗
Decrease (< 150)	18 (12.5)	35.22 ​**±** ​8.11		
Normal (150–350)	126 (87.5)	27.69 ​**±** ​9.60		
Increase (> 350)	0 (0.0)	0.00 ​**±** ​0.00		
ECOG fitness score (scores)			15.711^c^	< 0.001∗∗∗
0	4 (2.8)	16.50 ​**±** ​9.18		
1	46 (31.9)	22.96 ​**±** ​9.03		
2	55 (38.2)	29.09 ​**±** ​7.86		
3	28 (19.4)	35.07 ​**±** ​8.30		
4	11 (7.6)	38.09 ​**±** ​4.48		
5	0 (0.0)	0.00 ​**±** ​0.00		
Hospital anxiety and depression scale (scores)			6.476^c^	0.002∗∗
Normal (0–7)	43 (29.9)	30.15 ​**±** ​9.21		
Mild anxiety depression (8–10)	33 (22.9)	27.07 ​**±** ​7.48		
Moderate anxiety depression (11–14)	32 (22.2)	22.52 ​**±** ​11.06		
Severe anxiety depression (15–21)	36 (25.0)	28.63 ​**±** ​9.73		
Insomnia severity index (scores)			6.827^c^	< 0.001∗∗∗
No insomnia (0–7)	57 (39.6)	26.54 ​**±** ​10.89		
Mild insomnia (8–14)	49 (34.0)	27.04 ​**±** ​8.03		
Moderate insomnia (15–21)	32 (22.2)	32.53 ​**±** ​7.22		
Severe insomnia (22–28)	6 (4.2)	40.67 ​**±** ​9.61		
Family APGAR index (scores)			4.818^c^	0.009∗∗
Good family function (≥ 7)	92 (63.9)	27.04 ​**±** ​9.78		
Moderate family dysfunction (4–6)	38 (26.4)	30.18 ​**±** ​9.14		
Severe family dysfunction (≤ 3)	14 (9.7)	34.86 ​**±** ​8.22		
Patient-generated subjective global assessment (scores)			43.370^c^	< 0.001∗∗∗
Normal nutrition (0–3)	15 (10.4)	18.73 ​**±** ​12.03		
Moderate malnutrition (4–8)	56 (38.9)	23.82 ​**±** ​7.20		
Severe malnutrition (≥ 9)	73 (50.7)	34.36 ​**±** ​6.98		

‘a’ indicated the t-values not assuming equal variances; ‘b' indicated the *t*-values assuming equal variances; ‘c' indicated the *F*-values; ∗∗ indicates that the *P*-value < 0.01; ∗∗∗indicates that the *P*-value < 0.001. RA, refractory anemia; RAS, refractory anemia with ring sideroblasts; RCMD, refractory cytopenia with multilineage dysplasia; RCMRD, refractory cytopenia with multilineage dysplasia and increased ring sideroblasts; RAEB, refractory anemia with excess blasts; MDS-U, MDS unclassified; IPSS-R, Revised International Prognostic Scoring System; CHG regimen, recombinant human granulocyte colony-stimulating factor ​+ ​homoharringtonine ​+ ​cytarabine; ECOG, Eastern Cooperative Oncology Group; APGAR, Activity, Pulse, Grimace, Appearance, Respiration.

### Score on the scale for myelodysplastic syndromes patients

#### Scoring profile of cancer fatigue scale

The CFS scores of MDS patients were 28.63 ​± ​9.73, with a median and interquartile range of 30 (22–35). The average scores of each dimension from highest to lowest being: physical fatigue dimension score 16.76 ​± ​5.32, emotional fatigue dimension score 6.65 ​± ​2.90, and cognitive fatigue dimension score 5.22 ​± ​3.10.

#### Scoring profile of hospital anxiety and depression scale

The HADS scores were 10.38 ​± ​6.06, with the average scores of each dimension from highest to lowest being: depression dimension score 5.30 ​± ​3.58 and anxiety dimension score 5.08 ​± ​3.06. Among these, 43 cases (29.9%) were negative for anxiety and depression, while 101 cases (70.1%) were positive.

#### Scoring profile of insomnia severity index scale

The ISI scores were 9.99 ​± ​6.34. Among the patients, 57 (39.6%) were free from insomnia, while 87 (60.4%) suffered from insomnia.

#### Scoring profile of family APGAR index

The APGAR scores were 7.44 ​± ​2.67. Among the patients, 92 (63.9%) had good family functioning, while 52 (36.1%) had family dysfunction.

#### Scoring profile of patient-generated subjective global assessment

The PG-SGA scores were 8.44 ​± ​4.27. Among the patients, 15 (10.4%) had good nutritional status, while 129 (89.6%) were malnourished.

### Univariate analysis of cancer-related fatigue in myelodysplastic syndromes

Univariate analysis revealed significant associations (*P* ​< ​0.05) between CRF severity in MDS patients and multiple factors: age, disease subtype, IPSS-R risk stratification, number of chemotherapies, chemotherapy regimens (5-Azacitidine, CHG, and 5-Azacitidine ​+ ​CHG), treatment status, ECOG performance status, serum albumin, prealbumin levels, anxiety/depression, family support, insomnia, and nutritional status. Detailed results are presented in Table 1.

### Validation of treatment rationality of chemotherapy regimen and difference analysis of cancer-related fatigue

Cross-analysis of chemotherapy regimens and IPSS-R risk stratification showed that 81.9% of low-risk group patients and 91.8% of high-risk group patients received clinically guideline-recommended treatments (Table 2). Among high-risk patients, 90.2% received 5-Azacitidine therapy (vs. 15.7% in low-risk group, *P* ​< ​0.001), 77.0% received CHG regimen (vs. 13.3% in low-risk group, *P* ​< ​0.001), and 70.5% received 5-azacitidine ​+ ​CHG combination therapy (vs. 9.6% in low-risk group, *P* < ​0.001). One-way ANOVA results demonstrated significant differences in CRF scores among different chemotherapy regimens (*F* ​= ​6.753, *P* ​< ​0.001). Post-hoc tests indicated statistically significant differences only between “Whether to treat (No) vs. 5-Azacytidine” (mean difference ​= ​−8.56, *P* ​= ​0.013) and “Whether to treat (No) vs. 5-Azacytidine ​+ ​CHG regimen” (mean difference ​= ​−6.35, *P* ​= ​0.001) (Table 3).

**Table 2:** Association between Chemotherapy regimen and Revised International Prognostic Scoring System risk category.

Characteristics	Low-risk group	High-risk group	χ^2^	*P*-value
	*n* ​*=* ​83, *n* (%)	*n* ​= ​61, *n* (%)		
Guideline-treatment consistency			2.101	0.147
Concordant group	68 (81.9)	56 (91.8)		
Discordant group	15 (18.1)	5 (8.2)		
Chemotherapy regimen				
5-Azacytidine (yes)	13 (15.7)	55 (90.2)	75.342	< 0.001∗∗
CHG regimen (yes)	11 (13.3)	47 (77.0)	56.866	< 0.001∗∗
5-Azacytidine ​+ ​CHG regimen (yes)	8 (9.6)	43 (70.5)	54.294	< 0.001∗∗
Whether to treat (no)	68 (81.9)	5 (8.2)	73.55	< 0.001∗∗

∗∗indicates that the *P*-value < 0.01. CHG regimen, recombinant human granulocyte colony-stimulating factor ​+ ​homoharringtonine ​+ ​cytarabine.

**Table 3:** Post hoc comparisons (Tukey HSD) for cancer-related fatigue scores.

Chemotherapy regimen (I)	Chemotherapy regimen (J)	*N*	cancer-related fatigue scale scores	Mean difference (I-J)	*P*-value
			Mean ​± ​SD		
Whether to treat (no) vs.	CHG regimen	3	24.67 ​**±** ​2.31	0.76	0.999
	5-Azacytidine	13	34.00 ​**±** ​8.63	−8.56	0.013∗
	5-Azacytidine ​+ ​CHG regimen	55	31.80 ​**±** ​7.91	−6.35	0.001∗∗

∗indicates that the *P*-value < 0.05; ∗∗ indicates that the *P*-value < 0.01. CHG regimen, recombinant human granulocyte colony-stimulating factor ​+ ​homoharringtonine ​+ ​cytarabine; SD, standard deviation.

### Correlation analysis of cancer-related fatigue in patients with myelodysplastic syndrome

Pearson correlation analysis was employed to examine relationships between CRF and HADS, ISI, APGAR, PG-SGA scores, and number of chemotherapies in MDS patients. Results demonstrated significant positive correlations between CRF and HADS (*r* ​= ​0.539, *P* ​< ​0.01), ISI (*r* ​= ​0.360, *P* ​< ​0.01), PG-SGA (*r* ​= ​0.658, *P* ​< ​0.01), and number of chemotherapies (*r* ​= ​0.319, *P* ​< ​0.01), while a negative correlation was observed with APGAR (*r* ​= ​−0.249, *P* ​< ​0.01). Complete correlation data are presented in Table 4.

**Table 4:** Correlational analysis of cancer-related fatigue in patients with myelodysplastic syndromes (*N* ​= ​144).

Characteristics	Cancer-related fatigue	Hospital Anxiety and Depression Scale	Insomnia severity index	Family APGAR Index	Patient-Generated Subjective Global Assessment	The number of chemotherapies
Cancer-related fatigue	1.000					
Hospital anxiety and depression scale	0.539∗∗	1.000				
Insomnia severity index	0.360∗∗	0.549∗∗	1.000			
Family APGAR index	−0.249∗∗	−0.387∗∗	−0.333∗∗	1.000		
Patient-generated subjective global assessment	0.658∗∗	0.588∗∗	0.394∗∗	−0.273∗∗	1.000	
The number of chemotherapies	0.319∗∗	0.369∗∗	0.302∗∗	−0.152	0.332∗∗	1.000

∗∗indicates that the *P*-value < 0.01. APGAR, Activity, Pulse, Grimace, Appearance, Respiration.

### Multivariate linear regression analysis of influencing factors of cancer-related fatigue in myelodysplastic syndromes patients

Variables with statistical significance (*P* ​< ​0.05) from univariate and correlation analyses served as control variables. Hierarchical multiple linear regression, at an entry level of 0.05 and a removal level of 0.10, explored factors influencing cancer-related fatigue in MDS patients. The value assignment of independent and dummy variables is detailed in Table 5.

**Table 5:** Assignment of multivariate hierarchical regression independent variables and dummy variables.

Independent variable	Assignment specification
Age	"18–59" ​= ​1; "60–69" ​= ​2; "70–79" ​= ​3; ">80" ​= ​4
Disease classification	"RA" = (0, 0, 0, 0, 0, 0, 0, 0); "RAS" = (0, 1, 0, 0, 0, 0, 0, 0); "RCMD" = (0, 0, 1, 0, 0, 0, 0, 0); "RAEB-I" = (0, 0, 0, 1, 0, 0, 0, 0); "RAEB-II" = (0, 0, 0,0, 1, 0, 0, 0); "MDS-U" = (0, 0, 0, 0, 0, 1, 0, 0); (with RA group as reference, 5 dummy variables are generated)
IPSS-R risk classification	"Low-risk group" ​= ​0; "High-risk group" ​= ​1
5-Azacytidine	"No" ​= ​0; "Yes" ​= ​1
CHG regimen	"No" ​= ​0; "Yes" ​= ​1
5-Azacytidine ​+ ​CHG regimen	"No" ​= ​0; "Yes" ​= ​1
Whether to treat	"No" ​= ​0; "Yes" ​= ​1
The number of chemotherapies	"0" ​= ​1; "1–6" ​= ​2; "> 6" ​= ​3;
ECOG fitness score	"0" ​= ​0; "1" ​= ​1; "2" ​= ​2; "3" ​= ​3; "4" ​= ​4; "5" ​= ​5;
Albumin	"Decrease" ​= ​1; "Normal" ​= ​2; "Increase" ​= ​3;
Prealbumin	"Decrease" ​= ​1; "Normal" ​= ​2; "Increase" ​= ​3;
Hospital anxiety and depression scale	Plug in the original value
Insomnia severity index	Plug in the original value
Family care index questionnaire	Plug in the original value
Patient-generated subjective global assessment	Plug in the original value

RA, refractory anemia; RAS, refractory anemia with ring sideroblasts; RCMD, refractory cytopenia with multilineage dysplasia; RAEB, refractory anemia with excess blasts; MDS-U, MDS unclassified; IPSS-R, Revised International Prognostic Scoring System; CHG regimen, recombinant human granulocyte colony-stimulating factor ​+ ​homoharringtonine ​+ ​cytarabine; ECOG, Eastern Cooperative Oncology Group.

Model 1 demonstrates the influence of control variables on the dependent variable. Results revealed that age, ECOG performance status, IPSS-R risk category, and REAB-II subtype (with RA as reference) significantly predicted CRF in MDS patients (*P* ​< ​0.05), independently accounting for 50.2% of the total variance. In Model 2, which adjusted for variables from Model 1, we examined the independent variables' effects on the outcome. Anxiety-depression composite scores showed significant predictive effects on CRF (*β* ​= ​0.172, *P* ​< ​0.05), while subjective global nutritional status demonstrated a notable predictive influence (*β* ​= ​0.319, *P* ​< ​0.001), collectively explaining 62.5% of the total variance. Notably, anxiety and depression emerged as the most prominent contributors, independently explaining 12.3% of the variance as presented in Table 6.

**Table 6:** Multivariate linear hierarchical regression analysis of influencing factors of cancer-related fatigue in myelodysplastic syndromes patients (*N* ​= ​144).

Predictor	Model 1		Model 2	
	*β*	*t*	*β*	*t*
**(Constant)**	–	2.613	–	2.739
Age	0.260	3.276∗∗	0.178	2.490∗
RAS	0.086	0.899	0.102	1.196
RCMD	0.193	1.552	0.134	1.196
RAEB-I	0.232	1.862	0.163	1.460
RAEB-Ⅱ	0.276	2.275∗	0.165	1.516
MDS-U	−0.047	−0.660	−0.043	−0.661
IPSS-R risk classification	0.249	2.484∗	0.252	2.829∗∗
5-Azacytidine	−0.108	−0.365	−0.226	−0.861
CHG regimen	−0.355	−1.529	−0.490	−2.366∗
5-Azacytidine ​+ ​CHG regimen	−0.017	−0.084	0.025	0.141
Whether to treat	−0.312	−0.966	−0.482	−1.678
The number of chemotherapies	−0.010	−0.069	−0.042	−0.320
ECOG fitness score	0.332	4.043∗∗∗	0.151	1.854
Albumin	−0.150	−1.892	−0.119	−1.686
Prealbumin	0.007	0.087	0.045	0.650
Hospital anxiety and depression scale	–	–	0.172	2.088∗
Insomnia severity index	–	–	0.043	0.606
Family APGAR index	–	–	−0.047	−0.743
Patient-generated subjective global assessment	–	–	0.319	3.723∗∗∗
***R*^*2*^**	0.502		0.625	
**Adjusted *R*^*2*^**	0.444		0.567	
***ΔR*^*2*^**	–		0.123	
***F***	8.600∗∗∗		10.856∗∗∗	

∗ indicates that the *P*-value < 0.05; ∗∗ indicates that the *P*-value < 0.01; ∗∗∗ indicates that the *P*-value < 0.001; The fit of both models was significant (*P* ​< ​0.001). The variance inflation factor (VIF) values for all independent variables ranged from 1 to 4, indicating no multicollinearity among the variables. RAS, refractory anemia with ring sideroblasts; RCMD, refractory cytopenia with multilineage dysplasia; RAEB, refractory anemia with excess blasts; MDS-U, MDS unclassified; IPSS-R, Revised International Prognostic Scoring System; CHG regimen, recombinant human granulocyte colony-stimulating factor ​+ ​homoharringtonine ​+ ​cytarabine; ECOG, Eastern Cooperative Oncology Group.

## Discussion

### The status of cancer-related fatigue in patients with myelodysplastic syndromes

The study revealed a mean CRF score of 28.63 ​± ​9.73 (median 30, IQR 22–35) in Chinese MDS patients, indicating moderate fatigue levels yet notably lower than European cohorts reporting 30.60 ​± ​13.10 (*P* ​< ​0.05).^8^ This disparity may stem from multifactorial mechanistic underpinnings: 1) Cultural influences rooted in traditional stoicism might predispose Chinese patients to underrepresent symptom burden through self-assessment instruments; 2) Distinct cohort characteristics, as European populations included malignancy comorbidities – established CRF exacerbators.^29^ Dimensional analysis identified physical fatigue as the predominant manifestation (35.2% score distribution), pathophysiologically linked to MDS-associated cytopenias. Trilineage hematopoietic failure impairs tissue oxygenation capacity, directly compromising exercise tolerance – evidenced by significant hemoglobin-physical fatigue inverse correlation (*r* ​= ​−0.42, *P* ​= ​0.008), aligning with hematologic malignancy paradigms.^30^ This biopsychological synergy may establish a detrimental cycle: fatigue-impaired treatment adherence exacerbates therapy-related toxicities.^31^

CRF intervention strategies should prioritize hemoglobin optimization. Phase III trials demonstrate Traditional Chinese Medicine formulations (Yisui Granules/Shengxue Pills) elevate hemoglobin 1.5–2.0 g/dL (*P* ​= ​0.013) via CD34+ progenitor cell activation, correlating with 30% CRF reduction.^32^ These findings advocate integrated hematological rehabilitation models. Prospective studies should evaluate longitudinal CRF modulation through synergistic Eastern-Western therapeutic protocols.

### The impact of age on cancer-related fatigue in patients with myelodysplastic syndromes

Consistent with previous findings,^33^^,^^34^ this study demonstrates an association between CRF severity and age among Chinese MDS patients. The mean age of MDS patients was 61.33 ​± ​3.15, with those aged 70–79 and ​> ​80 years demonstrating greater CRF severity. This suggests a positive correlation between advancing age and CRF intensity. This progression may stem from age-related declines in physical resilience, psychological coping capacity, and functional independence among older patients. Concurrent psychosocial stressors—including financial strain, caregiver dependency concerns, and perceived familial burden—may amplify psychological distress. Such chronic stress manifests clinically as anxiety, irritability, and dysphoria, potentially disrupting HPA axis regulation and neurotransmitter homeostasis (e.g., serotonin and dopamine pathways), thereby exacerbating CRF.^35^ Furthermore, physiological deterioration in organ systems (particularly hematopoietic capacity and mitochondrial metabolic efficiency) naturally progresses with age, especially beyond 70 years, resulting in compromised energy production that directly intensifies CRF.^36^

The 2023 MDS therapeutic framework proposes age-stratified management using 70–75 years as a treatment threshold, which may optimize symptom control in elderly populations.^37^ However, the precise mechanisms underlying age-associated CRF exacerbation in elderly MDS patients remain unclear. Future longitudinal studies should investigate dynamic age-CRF interactions, particularly distinguishing disease progression effects from cumulative treatment toxicities. Development of geriatric-specific CRF assessment tools incorporating objective biomarkers (e.g., prealbumin, interleukin-6 [IL-6]) could enhance fatigue monitoring and facilitate timely therapeutic interventions to ameliorate CRF in this population.

### Chemotherapy exacerbates cancer-related fatigue severity in patients with myelodysplastic syndromes

The study cohort comprised 144 MDS patients, with 57.6% classified as low-risk and 42.2% as high-risk, mirroring recognized risk stratification patterns.^38^ While 81.9% of low-risk and 91.8% of high-risk patients received guideline-aligned therapies, 18.1% of low-risk patients underwent non-standard chemotherapy, including azacitidine or CHG regimens. This deviation may arise from guideline extensions permitting hypomethylating agents in transfusion-dependent low-risk patients unresponsive to supportive care, coupled with molecular genetic limitations in IPSS-R classification.^39^ Emerging IPSS-M criteria suggest molecular upstaging could reclassify some low-risk patients to higher-risk categories requiring intensive treatment.^40^

Azacitidine monotherapy recipients exhibited significantly heightened CRF severity compared to non-chemotherapy and combination therapy groups. As a cornerstone epigenetic therapy, azacitidine's DNA hypomethylation mechanism fundamentally differs from cytotoxic CHG regimens. Despite its efficacy in delaying AML progression and reducing transfusion dependence, prolonged administration may exacerbate fatigue through cumulative myelosuppression and metabolic alterations.^41^ Suboptimal response rates and inevitable disease progression in nearly half of treated patients highlight current biomarker limitations, with observed interethnic efficacy variations implying pharmacogenomic influences on therapeutic outcomes.^15^

Correlation analyses demonstrated a positive association between number of chemotherapies and CRF severity, corroborating findings by Michael et al.^42^ This pattern may reflect enhanced physiological stress from cumulative cytotoxic exposure.^43^ Notably, Donovan's team observed peak CRF intensification during initial treatment phases followed by stabilization,^44^ underscoring the need for rigorous fatigue assessment early in therapy. This study recommends implementing a stratified monitoring system combining pretreatment education with real-time symptom surveillance during induction therapy, followed by targeted supportive measures for persistent fatigue in maintenance phases. This approach aligns with evolving models of CRF progression while addressing physiological stress patterns associated with prolonged cytotoxic exposure.

### IPSS-R risk stratification impacts cancer-related fatigue severity in patients with myelodysplastic syndromes

The IPSS-R remains the gold standard for MDS prognostic stratification, demonstrating robust correlations with survival outcomes and AML transformation risks that dictate risk-adapted therapeutic strategies. Elevated IPSS-R risk stratification demonstrated significant associations with worsened prognosis and increased cancer-related fatigue severity, aligning with established observations.^38^ Current management prioritizes non-chemotherapeutic approaches for low-risk patients versus AML-type regimens for high-risk cases, despite their association with adverse outcomes including immunosuppression, cytotoxic metabolite accumulation, and proinflammatory cytokine release. Treatment-induced tissue damage elevates circulating mediators (C-reactive protein [CRP], IL-6, interleukin-1β [IL-1β], interferon [IFN], tumor necrosis factor-α [TNF-α] potentially mediating central fatigue through anemia and cachexia pathways.^45^

Notable cytogenetic disparities exist between elderly and younger MDS populations, with advanced age correlating with higher-risk IPSS-R profiles, accelerated progression, and clinical heterogeneity.^46^ These data emphasize the necessity of regular IPSS-R reassessment to detect disease evolution and optimize therapeutic interventions, particularly given age-related prognostic variations and treatment response differentials.

### Anxiety and depression exacerbate cancer-related fatigue in patients with myelodysplastic syndromes

Multivariate analysis identified HADS total scores as independent CRF predictors in MDS patients following demographic adjustment, with the depression subscale demonstrating superior predictive capacity over anxiety—consistent with hematologic malignancy fatigue models emphasizing depressive symptom centrality.^10^ While depression-fatigue causality remains incompletely elucidated, proposed pathophysiological mechanisms involve serotoninergic neurotransmission dysregulation and HPA axis dysfunction with associated endocrine disturbances.^5^ Clinically significant psychological distress manifests within three months post-diagnosis in nearly half of cancer patients, underscoring the inadequacy of single-timepoint evaluations.^47^ Implementation of baseline psychological assessment at diagnosis coupled with serial monitoring during this critical period is recommended, particularly for patients exhibiting rapid symptom progression or limited social support—enabling early identification of deteriorating psychoneuroimmunological interactions underlying CRF pathogenesis.

### Malnutrition exacerbates cancer-related fatigue in patients with myelodysplastic syndromes

Multivariate analysis confirmed PG-SGA scores as independent predictors of CRF severity, establishing nutritional status as a modifiable fatigue determinant consistent with evidence linking nutritional optimization to improved oncological outcomes.^48^ The pathophysiological interplay involves nutritional deficits impairing muscular bioenergetics through peripheral metabolic dysregulation, compounded by disproportionate n-6/n-3 PUFA ratios (OR ​= ​2.6, 95% CI: 1.2–5.5) promoting proinflammatory cascades.^5^^,^^49^ Clinically, targeted nutritional strategies such as glutamine-fortified formulations demonstrate dual therapeutic efficacy through gastrointestinal toxicity amelioration and immunocompetence enhancement, while adjuvant TCM regimens improve nutrient assimilation via splenic-gastric regulation in MDS management.^14^ These findings support multidimensional nutritional intervention systems integrating metabolic monitoring, inflammatory control, and immunometabolic homeostasis. Prospective investigations are warranted to delineate dose-response relationships between specific nutrients and CRF attenuation.

### Implications for nursing practice and research

Moderate levels of CRF in MDS patients demand targeted nursing interventions. Nurses should integrate a comprehensive assessment of patients' anxiety, depression, and nutritional status into routine care, as these factors are pivotal in influencing CRF. The fact that MDS itself contributes more to fatigue than chemotherapy side effects suggest a need for deeper exploration into the disease's impact on patients' energy levels and well-being.

Clinical nurses are poised to play a critical role in developing personalized care plans that address the multifaceted nature of CRF. By considering the unique conditions of each patient, medical staff can promote comprehensive strategies for intervening in MDS patients' anxiety and depression. Such as using narrative therapy and mindful Tai Chi to relieve fatigue and boost quality of life. Currently, no related research exists in this area for MDS patients, deserving further exploration. Through these strategies, nursing practice can significantly contribute to reducing the burden of CRF, thereby improving clinical outcomes and the overall well-being of MDS patients.

### Limitations

This study has several limitations inherent to its single-center cross-sectional design and restricted sample size, potentially limiting generalizability given the predominant elderly cohort and age-dependent treatment variability in MDS management. While standardized institutional protocols were maintained, international variations in treatment algorithms and healthcare policies may affect reproducibility. The convenience sampling approach introduces selection bias risks, warranting validation through multi-center longitudinal studies with experimental designs. Reliance on self-reported measures introduces potential reporting and recall biases, necessitating complementary objective biomarkers and third-party observational assessments. Although the CFS effectively characterized fatigue patterns, the absence of validated diagnostic thresholds for MDS-specific CRF precludes precise prevalence estimation. Future investigations should employ psychometrically validated instruments like the BFI for robust epidemiological characterization while establishing population-specific cutoff values through external validation cohorts.

## Conclusions

This study revealed moderate CRF levels in MDS patients, which nevertheless exerted significant detrimental effects on daily functioning. Multivariate analysis identified five independent risk factors: advanced age, high-risk IPSS-R classification, CHG chemotherapy regimens, comorbid anxiety-depression, and malnutrition. Notably, psychological distress and nutritional imbalance emerged as the most clinically modifiable core determinants. By delineating the multidimensional CRF risk profile in MDS patients—particularly the synergistic interplay between psychological and nutritional factors—this work provides novel evidence for hematological oncology nursing practice. We recommend integrating standardized fatigue assessment into routine care protocols and developing multimodal management frameworks combining psychological support with nutritional optimization.

## CRediT authorship contribution statement

**Libing Liang:** The author was primarily responsible for data collection, data entry, and manuscript preparation. **Jian Gao:** The author was primarily responsible for the data importation and analysis tasks. **Caiqin Wu:** Conceptualization, methodology, data curation, writing—review, and editing **Yan Qian:** Conceptualization, methodology, data curation, writing—review and editing, and supervision. All authors have read and approved the final manuscript.

## Ethics statement

This study was approved by the Ethics Committee of Shanghai Sixth People's Hospital [Approval No. 2022-KY-171(K)] and was conducted in accordance with the 1964 Helsinki Declaration and its later amendments or comparable ethical standards. All participants provided written informed consent.

## Funding

This work was supported by the Shanghai Jiao Tong University School of Medicine: Nursing Development Program (Grant No. SJTUHLXK2023). The funders had no role in considering the study design or in the collection, analysis, interpretation of data, writing of the report, or decision to submit the article for publication.

## Data availability statement

All the datasets of the current study are available from the corresponding author, CW, on reasonable request.

## Declaration of generative AI and AI-assisted technologies in the writing process

No AI tools/services were used during the preparation of this work.

## Declaration of Competing interest

The authors declare no conflict of interest.
