# Supplementary material for: The influencing factors of cancer-related fatigue in Chinese patients with myelodysplastic syndrome: A cross-sectional study
Source: Asia Pac J Oncol Nurs. 2025 May 2;12:100712. doi: 10.1016/j.apjon.2025.100712 (PMC12149548; doi:10.1016/j.apjon.2025.100712)
Supplement: Multimedia component 2 [file mmc2.docx]

**Appendix 1**

**General Information Survey**

亲爱的病友:

您好！首先感谢您对我院的信任，选择到我院进行治疗。我们全体医护人员衷心希望与您携手共抗病魔，并祝您早日康复！在疾病的治疗和康复中，您可能会因为一些身体或者心理上的不适而产生疲倦、倦怠或困惑等感受，从而影响您的生活质量。为了更加了解您目前的生理、心理及生活质量，我们诚挚地邀请您参加这次调查，请您认真如实填答这份问卷，让我们了解您目前的状况,以便进行更好的治疗与护理。

**1.性别:**口男 口女

**2.年龄：** 岁

**3.婚姻状况:** 口未婚 口已婚

**4.教育水平:** 口小学及以下 口初中 口高中/中专 口本科/大专 口 研究生

**5.家庭月收入**:口1000 元以下 口1000-3000 元

口3001-5000 元 口5001-10000元 口10000 元以上

**6.医疗费用支付方式**: 口自费 口医保

**7.骨髓异常增生综合征分型:**

口 难治性贫血（RA） 口 难治性贫血伴环状铁粒幼细胞（RAS）

口 难治性血细胞减少伴多系发育异常（RCMD） 口 5q-综合征

口 难治性贫血伴原始细胞过多Ⅰ型及Ⅱ型（RAEB-Ⅰ，RAEB-Ⅱ）

口 骨髓增生异常综合征，不能分型（MDS，不能分型）

**8. IPSS-R危险度分类：**

口极危: 1.5分； 口低危:＞1.5～3分； 口中危:＞3～4.5分；

口高危:＞4.5~6分；口极高危:＞6分

**9. 病程**：

**10. 化疗次数**： 次

**11. 5-阿扎胞苷（azacitidine,AZA）**：口有 口无

**12.去铁治疗：** 口有 口无

**13.ECOG体能评分：**

口 0 活动能力完全正常，与起病前活动能力无任何差异。

口 1 能自由走动及从事轻体力活动，包括一般家务或办公室工作，但不能从事较重的体力活动。

口 2 能自由走动及生活自理，但已丧失工作能力，日间不少于一半时间可以起床活动。

口 3 生活仅能部分自理，日间一半以上时间卧床或坐轮椅。

口 4 卧床不起，生活不能自理。。

口 5 死亡

**14．外周血白细胞计数**：

**15.血红蛋白：**

**16.血小板计数**：

**17.铁蛋白：**

**18.白蛋白：**

**19.前白蛋白：**

**20.BMI:**

**Appendix 2**

**Cancer Fatigue Scale (CFS)**

**1.容易疲劳吗？**

口 完全没有 口 极少 口 有一点 口 相当多 口 非常多

**2.想躺下来休息吗？**

口 完全没有 口 极少 口 有一点 口 相当多 口 非常多

**3.感到筋疲力尽吗？**

口 完全没有 口 极少 口 有一点 口 相当多 口 非常多

**4.觉得自己变得粗心了吗？**

口 完全没有 口 较少 口 有一点 口 相当多 口 非常多

**5.感到精力充沛吗？**

口 完全没有 口 极少 口 有一点 口 相当多 口 非常多

**6.身体有疲劳感吗？**

口 完全没有 口 极少 口 有一点 口 相当多 口 非常多

**7.觉得说错话的时候增多了吗？**

口 完全没有 口 极少 口 有一点 口 相当多 口 非常多

**8.对很多事情都感兴趣吗？**

口 完全没有 口 极少 口 有一点 口 相当多 口 非常多

**9.对什么都感到厌烦吗？**

口 完全没有 口 极少 口 有一点 口 相当多 口 非常多

**10.觉得自己变得健忘了吗？**

口 完全没有 口 极少 口 有一点 口 相当多 口 非常多

**11.做事情能集中注意力吗？**

口 完全没有 口极少 口 有一点 口 相当多 口 非常多

**12.觉得对什么都提不起劲吗？**

口 完全没有 口 极少 口 有一点 口 相当多 口 非常多

**13.觉得自己的思维变迟钝了吗？**

口 完全没有 口 极少 口 有一点 口 相当多 口 非常多

**14.能激励自己去做事情吗？**

口 完全没有 口 极少 口有一点 口 相当多 口 非常多

**15.疲劳无所适从吗？**

口 完全没有 口 极少 口 有一点 口 相当多 口 非常多

**Appendix 3**

**Hospital Anxiety and Depression Scale (HADS)**

**1、我感到紧张(或痛苦):**

0-根本没有( ) 1-有时候( ) ;

2-大多时候( ) 3-几乎所有时候()

**2、我对以往感兴趣的事情还是有兴趣:**

0-肯定一样( ) 1-不像以前那样多( )

2-只有一点( ) 3-基本上没有了( )

**3、我感到有点害怕好像预感到什么可怕的事情要发生:**

0-根本没有() 1-有一点，但并不使我苦恼( )

2-是有，不太严重( ) 3-非常肯定和十分严重( )

**4、我能够哈哈大笑，并看到事物好的一面:**

0-我经常这样( ) 1-现在已经不太这样了( )

2-现在肯定是不太多了( ) 3-根本没有( )

**5、我的心中充满烦恼:**

0-偶然如此( ) 1-时时，但并不轻松( )

2-时常如此( ) 3-大多数时间( )

**6、我感到愉快:**

0-大多数时间() 1-有时( ) 2-并不经常() 3-根本没有( )

**7、我能够安闲而轻松地坐着:**

0-肯定( ) 1-经常( ) 2-并不经常( ) 3-根本没有( )

**8、我对自己的仪容失去兴趣:**

0-我仍然像以往一样关心( ) 1-我可能不是非常关心( )

2-并不像我应该做的那样关心我( ) 3-肯定( )

**9、我有点坐立不安，好像感到非要活动不可:**

0-根本没有( ) 1-并不很少( ) 2-是不少( ) 3-却是非常多()

**10、我对一切都是乐观地向前看:**

0-差不多是这样做( ) 1-并不完全是这样( )

2-很少这样做( ) 3-几乎从不这样做( )

**11、我突然发现有恐慌感:**

0-根本没有( ) 1-并非经常( )

2-非常肯定，十分严重( ) 3-确实很经常( )

**12、我好像感到情绪在渐渐低落:**

0-根本没有( ) 1-有时( ) 2-很经常( ) 3-几乎所有时间( )

**13、我感到有点害怕，好像某个内脏器官变化了:**

0-根本没有() 1-有时() 2-很经常 ( ) 3-非常经常()

**14、我能欣赏一本好书或意向好的广播或电视节目:**

0-常常如此() 1-有时( ) 2-并非经常() 3-很少( )

**Appendix 4**

**Insomnia Severity Index (ISI)**

对于下面的每个问题，将最准确地描述您在过去两周中睡眠情况的选项数字打圈。

对于前三个问题，请评估您睡眠困难的严重程度。

**1. 难以入睡：**

无 轻微 中等 严重 非常严重

0 1 2 3 4

**2. 睡眠难以持续：**

无 轻微 中等 严重 非常严重

0 1 2 3 4

**3. 有清晨太早醒来的困扰：**

无 轻微 中等 严重 非常严重

0 1 2 3 4

**4. 您对目前睡眠情况的满意/不满意程度如何？**

非常满意 满意 意见中立 不满意 非常不满意

0 1 2 3 4

**5. 您认为您的睡眠问题影响您的日常生活运作（例如：白天的疲惫、做工作/日常杂务的 能力、专注的能力、记忆力、情绪）的程度如何？**

完全没影响 有点影响 相当影响 很大影响 极其影响

0 1 2 3 4

**6. 您的睡眠问题使您的生活品质受到影响，您认为其他人觉得这一情况的明显程度 如何？**

完全不明显 有点明显 相当明显 很明显 极其明显

0 1 2 3 4

**7. 对于您目前的睡眠问题，您感到担心/苦恼的程度如何？**

完全不担心 有点担心 相当担心 很担心 极其担心

0 1 2 3 4

把 7 题的数字加起来（问题 1+2+3+4+5+6+7） = _____________

**Appendix 5**

**Family APGAR Index (APGAR)**

填写下列问题，您就能对您的家庭有更好的了解，如果您对您的家庭成员或者本项目还有其他的补充，请写在补充说明处。“家庭”是指平常和您住在一起的成员，如果您是一个人居住，请将目前与您最密切的人当作您的家人。

| 问题 | 经常这样（2） | 有时这样（1） | 几乎很少（0） |
| --- | --- | --- | --- |
| 1.当我遇到问题时，可以向家人得到满意的帮助。 |  |  |  |
| 2.我很满意家人与我讨论各种事情以及分担问题的方式。 |  |  |  |
| 3.希望从事新的活动或发展时家人都能接受且给予支持。 |  |  |  |
| 4.我很满意家人对我表达感情方式以及对我的情绪的反应。 |  |  |  |
| 5.我很满意家人与我共度时光的方式。 |  |  |  |

**Appendix 6**

**Scored Patient-Generated Subjective Global Assessment (PG-SGA)**

**一、病人自评表**

1．体重

目前我的体重约为_______kg；1个月前体重约为_______kg；6个月前体重约为_____kg。身高:

在过去的2周，我的体重(工作表1）：

口减轻（1） 口没变化（0） 口增加（0）

工作表1　体重评分


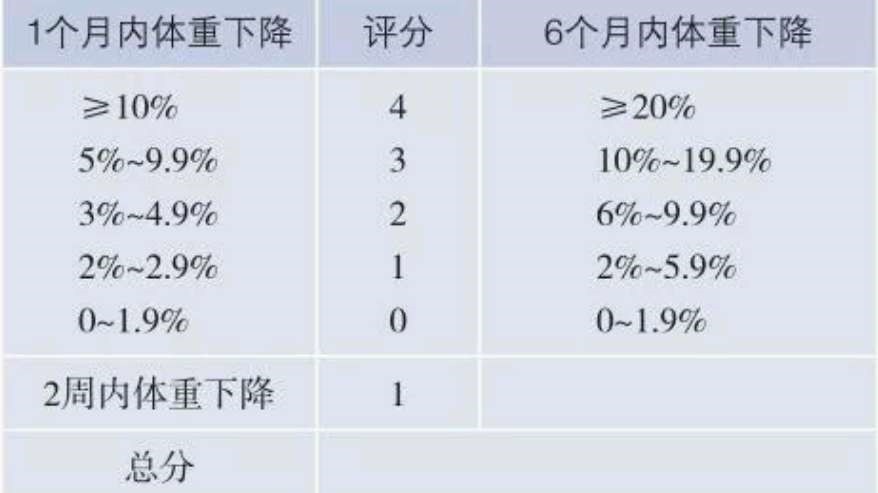


2．进食情况(**本项为多选，但是记分不做累加，以最高分选项为本项计分**)

在过去1个月里，我的进食情况与平时相比：

口没变化（0） 口比以往多（0） 口比以往少（1）

我目前进食：

口正常饮食（0） 口正常饮食，但比正常情况少（1）

口少量固体食物（2） 口只能进食流食（3）

口只能口服营养制剂（3） 口几乎吃不下什么（4）

口只能通过管饲进食或静脉营养（0）

3．症状

近2周来，我有以下问题，影响我的进食：

口吃饭没有问题（0） 口没有食欲，不想吃（3）

口恶心（1） 口呕吐（3） 口 口腔溃疡（2）

口便秘（1） 口腹泻（3） 口 口干（1）

口食品没味（1） 口食品气味不好（1） 口吞咽困难（2）

口一会儿就饱了（1）

口疼痛_________（部位）（3）

口其他__________（如抑郁，经济，牙齿）（1）

4．活动和身体功能

在过去的1个月病人的活动：

口正常， 无限制（0）

口不像往常，但还能起床进行轻微的活动（1）

口多数时候不想起床活动，但卧床或坐椅时间不超过半天（2）

口几乎干不了什么，一天大多数时候都卧床或在椅子上（3）

口几乎完全卧床，无法起床（3）

**二、医务人员评估表**

5．疾病与营养需求的关系**（B评分）**

相关诊断(特定）_____ 年龄_ __岁

原发疾病的分期： I II III IV其他（见工作表2）


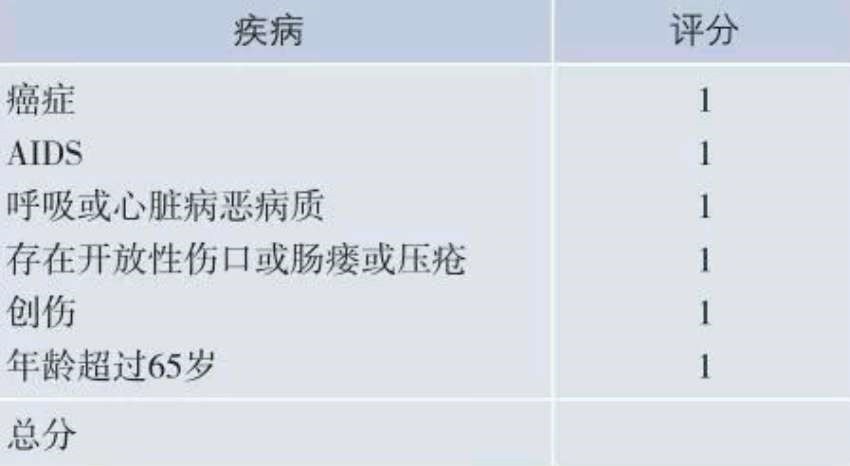


6．代谢方面的需求（应激状态）**（C评分）**

目前体温_____℃ 如果为发热，发热持续时间______小时；

是否用糖皮质激素 □ 是 药名__ _最大总剂量/天(mg）

□ 否(工作表3）

工作表3 　代谢方面的需求（应激评分）


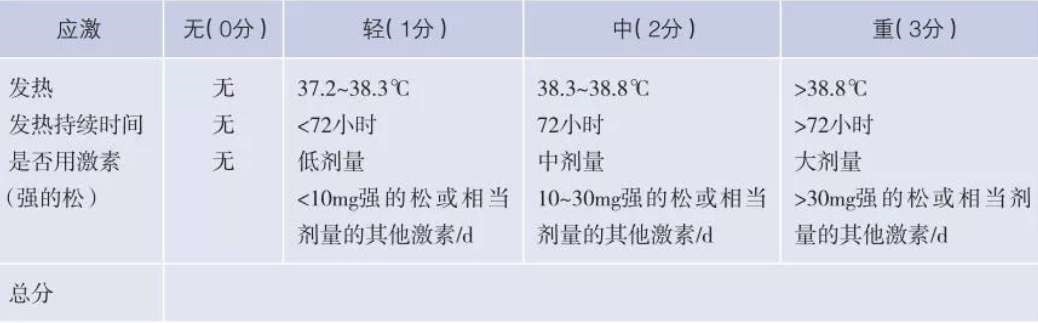


7．体格检查**（D评分）**

工作表4　体格检查表


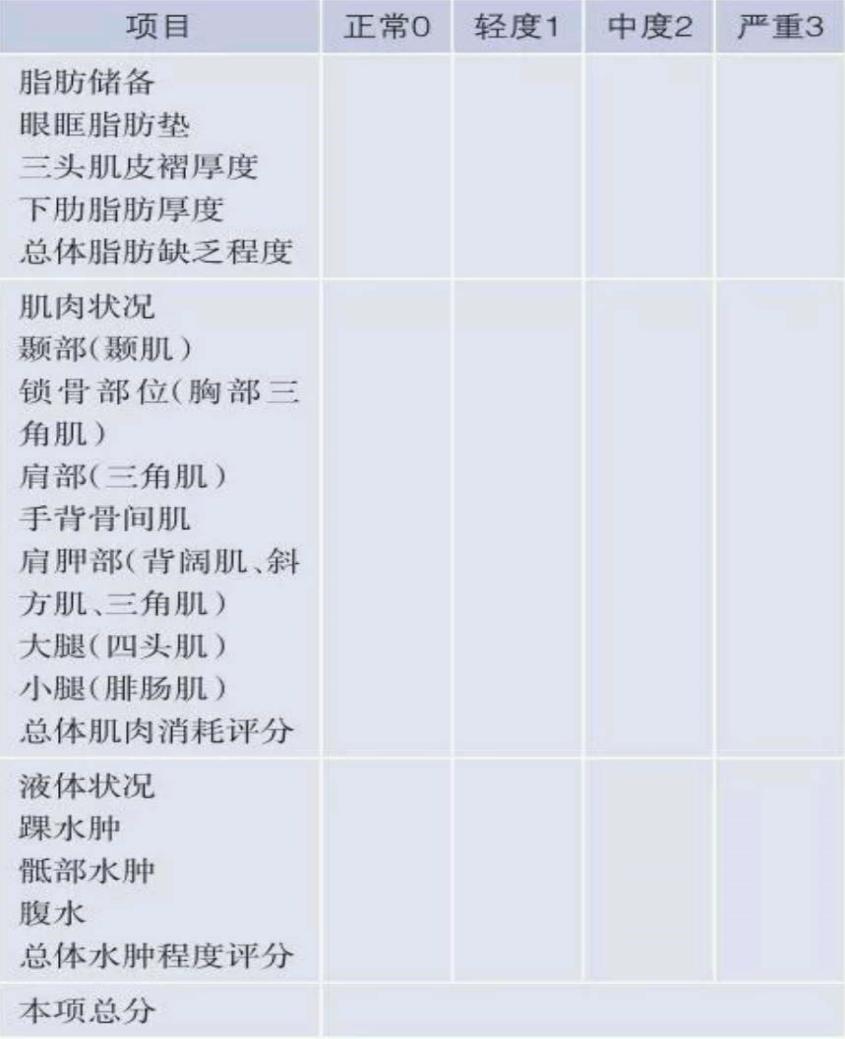


**Appendix 7**

**知情同意书**

尊敬的先生/女士：

您好！

我们是上海中医药大学护理学本科生，目前正进行有关骨髓增生异常综合征患者的癌因性疲乏现状及影响因素分析的研究，为更好的了解您目前健康状况及预后的情况，我们诚挚地邀请您参与我们的调查，您的参与和配合对本研究的顺利开展至关重要！

本研究无任何侵入性或创伤性的检查，仅通过调查问卷的形式进行评估。研究过程不会对您及您的家庭带来任何风险或伤害。主要研究过程包括：在您住院期间通过调查问卷对您进行身体和心理方面的评估，全程将花费您30分钟左右；并且我将全程在场，如果您有疑问可以与我交谈解决。

填写完问卷后，该研究数据将被编码，不会提及您的名字和泄露您的隐私。我们保证对您所提供的个人信息资料完全保密，不向外泄露。此项研究完全遵从您的意愿，您有权拒绝或中途退出，对您不会产生任何影响。感谢您的配合，祝您早日康复！

…………………………………………………………………………………………

我已经阅读这份同意书并自愿同意参与这项研究。

受调查人签字： 日期：______年____月____日

…………………………………………………………………………………………

我已向受调查者作详细解释并获得其同意参加本项研究。

调查者签字： 日期：______年____月____日
